# Importance of Considering Malnutrition and Sarcopenia in Order to Improve the QOL of Elderly Hemodialysis Patients in Japan in the Era of 100-Year Life

**DOI:** 10.3390/nu13072377

**Published:** 2021-07-12

**Authors:** Masaaki Inaba, Senji Okuno, Yoshiteru Ohno

**Affiliations:** 1 Department of Nephrology, Osaka City University Medical School, 1-4-3 Asahi-machi, Abeno-ku, Osaka 543-8585, Japan; 2 Kidney Center, Ohno Memorial Hospital, 1-26-10, Minami-Horie, Nishi-ku, Osaka 550-0015, Japan; teru11090116@yahoo.co.jp; 3 Kidney Center, Shirasagi Hospital, 7-11-23, Higashisumiyoshi-ku, Osaka 546-0002, Japan; okuno@shirasagi-hp.or.jp

**Keywords:** clinical malnutrition, older individuals, hemodialysis, sarcopenia, chronic kidney disease, quality of life, mortality

## Abstract

In the current aging society of Japan, malnutrition and resultant sarcopenia have been widely identified as important symptomatic indicators of ill health and can cause impairments of longevity and quality of life in older individuals. Elderly individuals are recommended to have sufficient calorie and protein intake so as to enjoy a satisfactory quality of life, including maintaining activities of daily living in order to avoid emaciation and sarcopenia. The prevalence of emaciation and sarcopenia in elderly hemodialysis (HD) patients in Japan is higher than in non-HD elderly subjects due to the presence of malnutrition and sarcopenia associated with chronic kidney disease (CKD). Furthermore, comorbidities, such as diabetes and osteoporosis, induce malnutrition and sarcopenia in HD patients. This review presents findings regarding the mechanisms of the development of these early symptomatic conditions and their significance for impaired QOL and increased mortality in elderly HD patients.

## 1. Introduction

**Figure 1:**
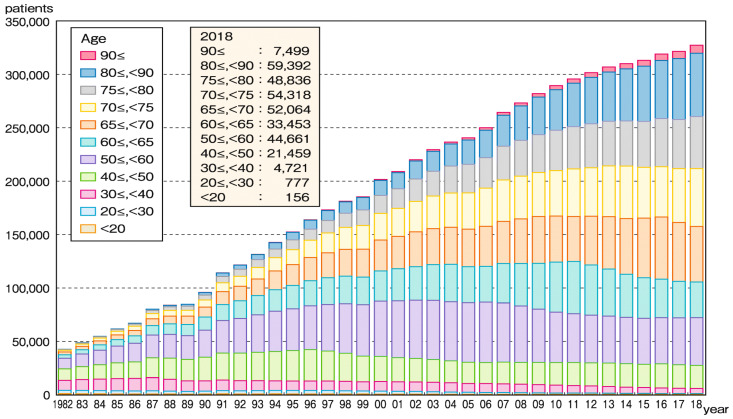
Trends in counts of Japanese hemodialysis patients stratified by age for the period 1982–2018. The proportion of patients aged ≥65 years and ≥75 years has increased up to 71% and 43%, respectively, in Japan. The average age of HD patients in Japan has remarkably increased during the last three decades, from 47 years old in 1983 to 69 years old in 2017 (registry of Japanese Society of Dialysis Therapy).

The society of Japan is aging, and the percentage of predialysis-chronic kidney disease (CKD) patients is greater in older populations; thus, it is not surprising that the number of elderly CKD patients who require renal replacement therapy (RRT) has been increasing. Since as few as 3% of dialysis patients can be maintained on peritoneal dialysis [1] and kidney transplantation is uncommon [2], nearly all end-stage CKD patients undergo hemodialysis (HD) as RRT. Moreover, over the last three-decade period, the average age of HD patients in Japan remarkably increased from 47 years in 1983 to 69 years in 2017, according to the registry of the Japanese Society of Dialysis Therapy (JSDT) [3]. In fact, the proportion of HD patients in Japan ≥65 years old has increased to 71%, and that of those ≥75 years old has increased to 43% (Figure 1) [4]. An analysis of the annual dialysis data report for the 2018 JSDT renal data registry [4] shows that the increasing age of HD patients in Japan can be accounted for by both elongation of HD duration due to the sophistication of dialysis techniques, and increasing age at the time of HD initiation. The DOPPS study demonstrated that the mortality rate in Japan is the lowest among the DOPPS-participating countries, and has continued to decline given the increasing age of Japanese HD patients. Along with the aging of the HD patient population, the number of co-morbidities that may impair a satisfactory quality of life (QOL), and thus cause emaciation and sarcopenia, is increasing. Impaired QOL in elderly HD patients changes patients’ condition from independent living to the requirement of physical support or nursing care [5], thus impairing the quality of a potential 100-year life. This review tries to elucidate the mechanism of the development of these presymptomatic conditions of emaciation/sarcopenia/frailty and their significance for impaired longevity, QOL, and mortality in elderly HD patients. Furthermore, the importance of diabetes and osteoporosis in the development of malnutrition and sarcopenia is emphasized.

## 2. Preferential Occurrence of Malnutrition and Its Mechanism in Pre-Dialysis CKD Patients

Old age is known to be associated with poor nutritional status, while CKD itself is closely associated with malnutrition because of several different mechanisms [6]. First, CKD may be associated with dietary inadequacy in relation to suboptimal energy and protein intake due to poor appetite status, taste perception [7], low diet quality, and/or psychosocial or financial barriers. Furthermore, a reduction in metabolic rate resulting from reduced physical activity and muscle mass can contribute to poor appetite in HD patients [8]. Second, a protein-restricted diet has been recommended for pre-dialysis CKD patients to prevent exacerbation of renal dysfunction, though that might be a risk factor for malnutrition and sarcopenia [9], which is supported by the findings showing the beneficial effects of a high-protein diet or amino acid supplementation on nutritional state, as assessed by improved serum albumin and various nutritional markers [10]. Third, CKD is complicated by a metabolic syndrome termed malnutrition–inflammation complex syndrome (MICS) [11], or protein energy wasting (PEW) syndrome [12], which consist of catabolic inflammatory reactions and cachexia leading to malnutrition. Fourth, it is possible that multiple comorbidities associated with malnutrition and frailty/sarcopenia, such as diabetes mellitus (DM), cardiovascular disease (CVD), cerebrovascular disease, immobility, and insomnia, contribute to malnutrition. Interestingly, a study of common malnutrition in pre-dialysis CKD patients found that more than 50% of nephrologists initiate dialysis for end-stage CKD patients after their nutritional status is impaired [13].

## 3. Significance of Malnutrition for Various Clinical Outcomes in HD Patients

Along with the common occurrence of malnutrition in end-stage CKD cases, a malnutritional state can continue even when the patient reaches a stable condition on maintenance HD. Although HD initiation allows for a more liberal intake of protein and food so as to improve nutritional state [14,15], dialysis-specific factors still exist that cause malnutrition. These relate to the low adequacy induced by 4-h HD sessions performed three times a week, which causes a persistent uremic state [16], metabolic acidosis [17], and the accumulation of various uremic substances in serum that disturb metabolism. A related study showed that patients in Japan who underwent extended-time HD sessions, based on a treatment policy of extending dialysis time and removing dietary restrictions, exhibited better survival, along with the maintenance of or an increase in body mass index [18]. Furthermore, a massive loss of nutrients and amino acid from circulation to dialysate via the high-performance dialysis membrane, as well as hemodiafiltration, are dialysis-specific mechanisms of malnutrition [19].

At the time of HD initiation, a patient presenting with malnutrition has a high mortality risk on the basis of low nutrition markers, such as geriatric nutritional risk index (GNRI) [20], subjective global assessment [21], low body mass index (BMI) [22], low serum levels of albumin [20] and cholesterol [21], and low food intake [23]. Among these parameters, we reported the clinical utility of GNRI as a relevant predictor for mortality in HD patients [20]. A GNRI value <90 was associated with a significantly lower survival rate in HD patients as compared to those with GNRI ≥90 [24]. Furthermore, we previously reported that HD patients who gained fat mass after HD initiation exhibited a better survival rate than those with loss of fat mass after HD initiation [25], and that fat mass gain after HD initiation was significantly associated with reductions in serum CRP, a reliable marker for inflammation and CVD risk. This suggests that the improvement of nutritional status might lead to the suppression of inflammation and atherosclerosis [26], and thus finally a better survival rate.

## 4. Significance of Sarcopenia in Relation to Harmful Effects of Malnutrition in HD Patients

Sarcopenia was defined in 1988 as an age-related reduction in skeletal muscle mass and function [27], after which the Asia Working Group for Sarcopenia provided a definition for the evaluation of sarcopenia in Asian individuals [28]. Serum albumin, which is reported to be elevated by an increased intake of food, protein, and branched-chain amino acids, also rises with an increase in muscle content. Furthermore, GNRI, a relevant marker for nutrition and mortality, is defined via serum albumin in addition to body weight, which is mainly determined by muscle content. We examined the importance of the creatinine index, another nutritional marker in HD patients without residual renal function, as a predictor of mortality risk [29]. The creatinine index is calculated using the following formula: Cr index = 16.21 ( +1.12 if male) − 0.06 × [age (years] − 0.08 × (single pool Kt/V) + 0.009 × [serum creatinine (μmol/L]. Thus, the creatinine index is a nutritional marker that is mainly determined in HD patients by muscle content, given the lack of apparent residual renal function. We found that lower GNRI and Cr index values were both independently and equally associated with an increased risk of all-cause mortality in a multivariable-adjusted model [29]. Taken together, these findings demonstrate that the mechanism by which malnutrition increases mortality risk in Japanese HD patients can be mostly explained by reduced muscle mass. Therefore, we next focused on the significance of the development of sarcopenia in HD patients.

## 5. Preferential Occurrence of Sarcopenia, and the Significance of Muscle Strength Rather Than Muscle Mass in HD Patients

Based on our report [30], with the increasing age of Japanese HD patients, the prevalence of sarcopenia among them was found to have increased to as much as 40% (37% in males and 45% in females). Although the definition of sarcopenia is based on muscle mass measurements by the Asian Working Group for Sarcopenia [28], it remains to be determined whether muscle mass or muscle strength is more important in determining the clinical outcome of sarcopenia in humans. Our study found that serum creatinine has a significantly positive correlation with not only muscle mass, determined via dual-energy X-ray absorptiometry (DXA), but also muscle strength measured by handgrip strength [31]. Therefore, we examined whether muscle mass or muscle strength might be a more important determinant of serum creatinine level in HD patients. Multivariate analysis demonstrated that poor arm muscle quality, calculated using the handgrip strength/DXA-determined arm lean mass ratio, rather than reduced DXA-determined arm lean mass, is responsible for the reduction in serum creatinine in HD patients [32]. This indicates that muscle strength is a more important factor than muscle mass as a determinant for serum creatinine level in HD patients. To confirm the harmful effects of reduced muscle strength on mortality in HD patients in Japan, we also examined the effects of impaired muscle quality, assessed by the reduced muscle strength/muscle mass ratio [31]. A total of 272 HD patients were divided into two equal-sized groups (higher and lower) based on muscle quality, and the Kaplan–Meier analysis results demonstrated that the higher group exhibited a significantly lower mortality rate than the lower group. Furthermore, Cox regression hazards analysis identified higher muscle quality as a significant independent predictor for survival in HD patients, independently of the presence of DM, age, and serum albumin level. In another study, higher age, female gender, longer HD duration, presence of DM, lower BMI, and higher CRP were shown to be independent factors associated with lower handgrip strength in HD patients [33]. Our recent findings also suggest that the efficient utility of ketone bodies, which are mainly utilized as an efficient energy source in the muscle tissues of HD patients, is an independent determinant of higher levels of albumin and uric acid in serum [34]. Serum albumin [35] and uric acid [36] are both established as nutritional markers intimately associated with mortality in HD patients. Furthermore, it was reported that a higher level of serum β-hydroxybutyrate, probably due to its impaired metabolism in muscle tissues, was independently associated with CVD events and all-cause mortality in HD patients [37]. Together, these findings indicate that a better energy metabolism in the muscle tissues of HD patients is important to maintaining whole body nutritional state and increasing survival, supporting the importance of muscle mass/strength for maintaining nutritional status and thus a better survival rate in HD patients.

## 6. DM and Sarcopenia in HD Patients

In addition to aging and malnutrition, sarcopenia is known to preferentially occur in HD patients with osteoporosis and DM [38]. Additionally, the rates for the co-existence of sarcopenia, osteoporosis, and DM are known to be higher in HD patients and increase with aging. Although each disease is known to independently affect physical activity and mortality in HD patients, it is possible that DM and osteoporosis, both independently and together with sarcopenia, might reduce longevity and survival rates in these patients. Furthermore, the interaction between these three diseases is important to mention.

The number of DM patients in aged populations is increasing [39]. In Asia, the prevalence of sarcopenia in type 2 (T2) DM has been shown to progressively increase with age (17.4%, 28.1%, 52.4%, and 60% in individuals aged 65–69, 70–74, 75–80, and >80 years, respectively) [40]. Additionally, a study conducted in Japan showed the prevalence of sarcopenia in T2DM patients who were ≥80 years old to be over 40% [41]. Since nearly all DM patients suffer from T2DM, but not T1DM, in Japan, DM patients who we previously examined exclusively had T2DM. A recent meta-analysis confirmed that the prevalence of sarcopenia is significantly higher in T2DM than non-DM patients [42]. It is known that serum creatinine levels are significantly lower in DM as compared to non-DM HD patients without residual renal function, which is consistent with our finding that DM HD patients exhibit significantly lower muscle mass and strength than their non-DM counterparts [31,33,34], and that lower handgrip strength is significantly associated with the presence of T2DM in HD patients [33]. To avoid the confounding effect of DM on the association between lower muscle quality and higher mortality rate, we examined the association between these two parameters separately in HD patients with and without DM [31], and those with lower muscle quality (both non-DM and DM patients) exhibited significantly higher mortality rates, indicating poor muscle quality as a significant and independent factor contributing to the higher mortality both in DM and non-DM HD patients. This may also suggest that the mechanism of increased mortality in HD patients with DM is due, at least in part, to poor muscle quality induced by a sustained DM state.

**Figure 2:**
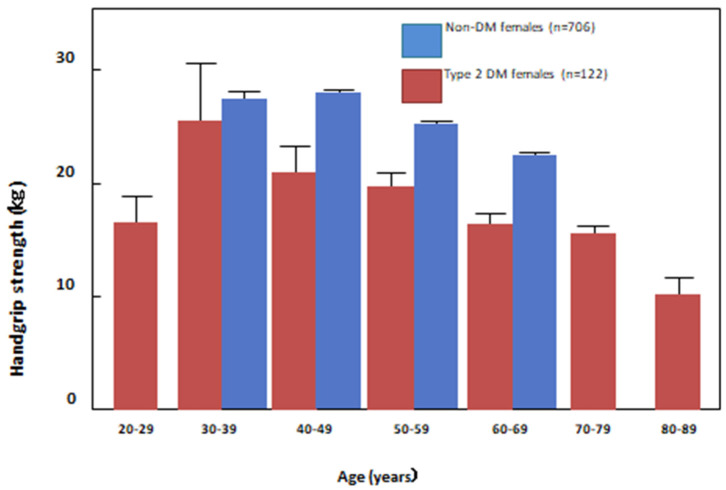
Age-stratified handgrip strength in normal female subjects and type 2 DM patients without overt DM complications. Although handgrip strength started to decrease significantly during the postmenopausal period as compared to normal female subjects, female type 2 diabetes patients exhibited a significant decrease in handgrip strength by their 40 s, supporting the notion that type 2 DM is a risk factor for the early development of muscle strength reduction.

To elucidate the association between DM alone with muscle strength independent of CKD, we measured handgrip strength in female T2DM patients without clinically overt DM complications in our DM outpatient clinic, and compared the results with those of a non-DM normal female control group of the same age [38]. Figure 2 shows the changes in handgrip strength with age in those female subjects. While non-DM female normal controls exhibited a characteristic decline in handgrip strength after menopause because of loss of estrogen, which has a protective effect on muscle [43], handgrip strength was significantly weaker in female DM patients in their 40 s than in their non-DM counterparts, which supports our finding in HD patients that DM is an independent risk factor for the development of sarcopenia in HD patients [31].

**Figure 3:**
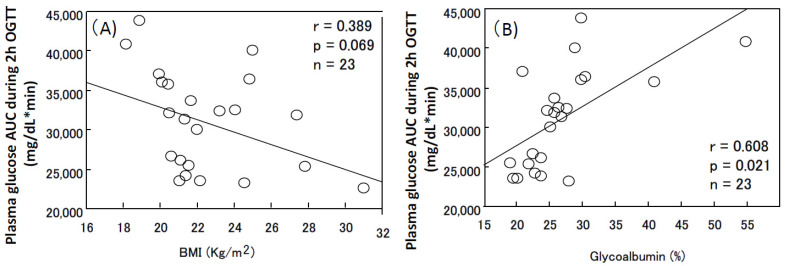
Correlation between area under curve (AUC) of plasma glucose during 2 h oral glucose tolerance test with BMI (**A**) and glycoalbumin (**B**) in hemodialysis patients. Oral glucose (75 g) tolerance test was performed in 23 Japanese hemodialysis patients after an overnight fast. The plasma glucose AUC during the 2 h oral glucose tolerance test exhibited a significant negative correlation with BMI (**A**) and a positive correlation with glycoalbumin (**B**).

A DM state has been shown to be associated with sarcopenia via several different mechanisms, including malnutrition, insulin/IGF-1 deficiency, and a sustained hyperglycemia condition, while it has been speculated that sarcopenia might exacerbate the DM condition because of reduced muscle tissue, against which insulin treatment protects by stimulating transport plasma glucose into muscle tissue. A study found that the energy intake of DM patients with sarcopenia, often observed in elderly DM patients, is significantly lower than that in sarcopenia-free DM patients [44]. Furthermore, energy intake in DM patients in that study was independently and negatively associated with sarcopenia, after adjustments for age, gender, exercise, smoking habit, HbA1c, and BMI. Since physical activity determines the metabolic rate associated with food intake, it is possible that DM HD patients with sarcopenia undertake less physical activity. In fact, DM prevalence in HD patients with a history of falling was significantly greater compared to those without such a history. The former group of patients also had lower serum levels of albumin and creatinine, and lower physical function test scores [45], suggesting an association between low physical performance and poor nutrition with prevalence of DM in HD patients. Additionally, the postprandial secretion of insulin has been shown to stimulate muscle/adipose tissue blood flow and have a musculotrophic effect that stimulates the cellular uptake of amino acids to induce de novo protein synthesis in muscle tissue [46]. Conversely, in individuals with relative or absolute insulin/IGF-1 deficiency, amino acids are lost from the muscle. Other major mechanisms of muscle injury are a sustained high-glucose condition [47] and broad glucose fluctuation [48]. Since glucose fluctuation is mainly induced by postprandial glucose excursion, which is suppressed by postprandial insulin secretion to enhance glucose entry into muscle tissue, sarcopenia alone presumably induces a greater increase in plasma glucose after consumption of a meal, which might further deteriorate muscle tissue given the increased oxidative stress generated by the increase in postprandial glucose. Indeed, the plasma glucose area under the curve during the 2 h oral glucose test of DM HD patients, which represents the increase in postprandial glucose (evidenced by a significant correlation with glycoalbumin, a clinically reliable marker for postprandial hyperglycemia [49]), exhibited a tendency towards inverse correlation with BMI, although this was not significant (Figure 3). These data also suggest that the maintenance of BMI, which is particularly affected by lean mass in HD patients, might protect postprandial glucose excursion in such patients.

Furthermore, hyperglycemia is a result of cellular malnutrition, given the incapability of glucose to enter muscle cells, leading to loss of muscle mass and the development of sarcopenia. DM complications, such as CVD, visual dysfunction, and dementia, can restrict physical activity, leading to loss of muscle tissue. Peripheral arterial disease, another complication often observed in DM HD patients, might also cause loss of muscle tissue by limiting the blood flow to the muscle tissue in the lower limbs [50].

## 7. Osteoporosis and Sarcopenia in HD Patients

We recently reported that pre-dialysis CKD patients with a fracture exhibited a greater creatinine-based eGFR/cystatin C-based eGFR ratio than those without a fracture [51]. Creatinine-based eGFR is known to overestimate true GFR in aged CKD patients, apparently because of the lower levels of serum creatinine resulting from reduced muscle mass, as observed in HD patients, and it has been shown that cystatin C-based eGFR reflects true GFR more effectively than creatinine-based eGFR in aged CKD patients [52]. Additionally, our results indicate that the fracture rate in pre-dialysis CKD patients is greater in those with than without sarcopenia [51]. Due to the high prevalence of sarcopenia in HD patients in Japan [30], it is reasonable to consider the importance of sarcopenia in the development of osteoporosis and osteoporosis-based fragility fractures. Fall trauma and fracture are amongst the main causes of changes in the conditions of elderly HD patients, from independent living to the requirement of physical support or nursing care. It is known that mechanical force to bone tissue stimulates bone formation, resulting in increased bone mineral density. We reported an association of handgrip strength with cortical thickness, but not with trabecular bone mineral density, at the 5.5% distal radius in both normal and type 2 DM subjects [38]. Thus, mechanical force generated by muscle contractions might exert a preferential effect on cortical bone components, a major determinant of bone strength in appendicular bones such as the femur [53]. Furthermore, sarcopenia is known to be a risk factor for femoral neck fracture because of the increased risk of falling [54,55] and the greater impact on the femur bone during a fall caused by the loss of cushioning provided by the gluteus maximus muscle [56]. Furthermore, HD patients with sarcopenia exhibit a higher risk of falling-induced fragility fractures [45,57]. Therefore, CKD should be regarded as a condition that increases the risk of femoral fracture due to the frequent occurrence of sarcopenia in affected individuals.

Although mechanical loading is a key mechanism that links bone and muscle, as mentioned above, the effects of muscle–bone interactions between two organs via secretome secretion have recently been emphasized [58]. Skeletal muscle tissues secrete chemical substances that have effects on bone metabolism, such as insulin, IGF-1, myostatin [59], basic fibroblast growth factor 2, IL-6, IL-15, osteoglycin, and osteoactivin. Additionally, the chemokines expressed by bone tissues potentially affect muscle metabolism, since osteocytes secrete prostaglandin E2 and Wnt3a, osteoblasts secrete osteocalcin and IGF-1, and both cell types produce sclerostin.

Since phosphate exists in bones as a form of hydroxylapatite, the stimulation of bone resorption via secondary hyperparathyroidism increases the degree of phosphate release from bones into the circulation in CKD patients. It is widely recognized that too much phosphate induces premature aging by stimulating atherosclerotic changes, renal damage, and osteoporosis [60], suggesting premature aging in CKD patients via stimulation of bone resorption. Indeed, previous studies have demonstrated an accelerated increase in acute myocardial infarction and cerebral infarction in postmenopausal women [61,62], and increased intima-media thickness of the common carotid artery and atherosclerotic plaque in middle-aged postmenopausal women compared to premenopausal women of the same age [63]. Furthermore, it was reported that coronary arterial calcification in postmenopausal women was advanced in those with osteoporosis as compared to those without [64], and that postmenopausal women with higher bone turnover exhibited higher mortality than those with lower bone turnover [65]. Other reports also demonstrated that higher serum phosphate may promote CKD progression, and attenuate the renoprotective effects of a low-protein diet and angiotensin-converting enzyme inhibitors in CKD patients [66,67]. We previously reported that higher bone turnover was correlated in a positive manner with higher urinary albumin excretion in postmenopausal women, but not in premenopausal women [68], which suggests the importance of the greater rate of phosphate release from bones caused by increased bone resorption during the development of renal damage in postmenopausal women. Additionally, a series of studies, including ours, confirmed the notion that the increased phosphate released from bones into the circulation, as a result of stimulated bone resorption, causes cardiovascular and renal damage in postmenopausal osteoporotic patients, given the protective effects of bone anti-resorptive drugs, such as denosumab [69] and bazedoxifene [70], on renal function in female osteoporotic patients. Furthermore, it was shown that, in osteoporotic patients with and without bone anti-resorptive drug treatment, those with increased bone mineral density exhibited improved pulse wave velocity (an early marker of arterial wall sclerosis) and carotid artery intima-media thickness (an early marker of arterial wall thickening) [71]. Finally, the administration of bisphosphonate, a potent anti-resorptive agent, was demonstrated to suppress the incidence of acute myocardial infarction in osteoporotic patients [72]. Together, these findings clearly demonstrate that enhanced osteoporosis-associated bone resorption enhances premature aging in vessels and kidneys by increasing the phosphate release from bones.

## 8. Sarcopenia as a Risk for Mortality and Impaired QOL in HD Patients

In our study that examined the effects of muscle quality on mortality in HD patients in Japan, Kaplan–Meier analysis showed that those with higher muscle quality had a lower mortality rate than those with lower muscle quality [31]. Additionally, Cox regression hazards analysis identified greater muscle quality as a significant independent predictor for better survival in our Japanese HD patients (HR: 0.889, 95% CI 0.814–0.971; *p* < 0.05) after adjustments for age, sex, and prevalence of DM. Previous findings also demonstrate the association between lower muscle quality and impaired physical performance [54]. We consider that the maintenance of muscle quality should be recognized as a clinically important target to elongate the life span and maintain the QOL of HD patients.

## 9. Importance of Sarcopenia as a Treatment Target to Elongate Longevity of HD Patients in the Era of 100-Year Life

As written above, it seems that various co-morbidities preferentially existing in HD patients, such as diabetes, pretexting CVD, fracture, and malnutrition, can impair longevity and life quality in older HD patients, in part via sarcopenia. Although the main cause of sarcopenia might differ between HD patients, it is now increasingly being identified as an early symptomatic indicator of ill health in elderly people, and thus is a definite target for prevention and treatment in order to elongate longevity in HD patients in the era of 100-year lives. HD patients with sarcopenia, which is often accompanied with malnutrition, are strongly encouraged to maintain sufficient calorie and protein intakes so as to enjoy a satisfactory quality of life, which includes maintaining the activities of daily living that will help to avoid and or reverse emaciation and sarcopenia. However, efforts to increase food intake in HD patients with sarcopenia/emaciation often are not successful due to persistent anorexia resulting from sarcopenia-associated inflammatory status [73]. The first step to stop this vicious cycle should be physical therapy. It is possible that physical therapy might increase appetite by lifting the patient’s mood in the short term, and increasing the metabolic rate via activated muscle metabolism/mass in the long term. Since the Japanese Ministry of Health, Labour and Welfare recommends a high-calorie and high-protein diet to increase longevity in the elderly population, this food policy should be extended to elderly HD patients, after encouraging them to undertake physical therapy.
